# LFP Oscillations in the Mesencephalic Locomotor Region during Voluntary Locomotion

**DOI:** 10.3389/fncir.2017.00034

**Published:** 2017-05-19

**Authors:** Brian R. Noga, Francisco J. Sanchez, Luz M. Villamil, Christopher O’Toole, Stefan Kasicki, Maciej Olszewski, Anna M. Cabaj, Henryk Majczyński, Urszula Sławińska, Larry M. Jordan

**Affiliations:** ^1^The Miami Project to Cure Paralysis, Department of Neurological Surgery, University of Miami Miller School of MedicineMiami, FL, United States; ^2^Department of Neurophysiology, Nencki Institute of Experimental BiologyWarsaw, Poland; ^3^Department of Physiology, Spinal Cord Research Centre, University of ManitobaWinnipeg, MB, Canada

**Keywords:** deep brain stimulation, mesencephalic locomotor region, local field potentials, locomotion, spinal cord injury

## Abstract

Oscillatory rhythms in local field potentials (LFPs) are thought to coherently bind cooperating neuronal ensembles to produce behaviors, including locomotion. LFPs recorded from sites that trigger locomotion have been used as a basis for identification of appropriate targets for deep brain stimulation (DBS) to enhance locomotor recovery in patients with gait disorders. Theta band activity (6–12 Hz) is associated with locomotor activity in locomotion-inducing sites in the hypothalamus and in the hippocampus, but the LFPs that occur in the functionally defined mesencephalic locomotor region (MLR) during locomotion have not been determined. Here we record the oscillatory activity during treadmill locomotion in MLR sites effective for inducing locomotion with electrical stimulation in rats. The results show the presence of oscillatory theta rhythms in the LFPs recorded from the most effective MLR stimulus sites (at threshold ≤60 μA). Theta activity increased at the onset of locomotion, and its power was correlated with the speed of locomotion. In animals with higher thresholds (>60 μA), the correlation between locomotor speed and theta LFP oscillations was less robust. Changes in the gamma band (previously recorded *in vitro* in the pedunculopontine nucleus (PPN), thought to be a part of the MLR) were relatively small. Controlled locomotion was best achieved at 10–20 Hz frequencies of MLR stimulation. Our results indicate that theta and not delta or gamma band oscillation is a suitable biomarker for identifying the functional MLR sites.

## Introduction

Several areas of the brain have been shown to elicit locomotion when stimulated, including areas of the diencephalon and mesencephalon (Grillner et al., 1997; Jordan and Sławińska, 2014; Kiehn, 2016; Takakusaki et al., 2016). Deep brain stimulation (DBS) of the mesencephalic locomotor region (MLR), a coordination center for activation and control of spinal locomotor generator neurons, is increasingly under consideration as a potential treatment strategy for improving locomotion in Parkinson’s disease (PD; freezing of gait, or FOG) and following spinal cord injury (SCI). The MLR was originally described in the cat by Shik et al. (1966), and was considered to be co-extensive with the cuneiform nucleus (CnF). This conclusion has been confirmed by many researchers in subsequent years, and this evidence has been extensively reviewed (Mori et al., 1989, 1992; Whelan, 1996; Grillner et al., 1997; Jordan, 1998; Ryczko and Dubuc, 2013; Jordan and Sławińska, 2014). The pedunculopontine nucleus (PPN) is considered a component of the MLR, a suggestion advanced by Garcia-Rill et al. (1986, 1987, 2011). They pointed out that the PPN is defined by the presence of cholinergic neurons, and gait defects, especially in PD, have been proposed to be due to pathological changes in the cholinergic neurons in the PPN. As a result, attempts to localize the areas suitable for DBS for improving locomotion by stimulating the MLR area have focused on the PPN, but increasingly it has been recognized that the CnF and associated structures may be more appropriate targets (Mazzone et al., 2005; Stefani et al., 2007; Piallat et al., 2009; Shimamoto et al., 2010; Alam et al., 2011; Hamani et al., 2011; Thevathasan et al., 2012).

Local field potentials (LFPs) can serve as biomarkers for movement disorders and for improved electrode targeting for DBS (Thompson et al., 2014), and the increasing emphasis on MLR DBS for restoring gait in human subjects necessitates the demonstration of LFPs recorded from effective MLR sites. Surprisingly, no study has been carried out on LFPs recorded in the functionally defined MLR under controlled conditions in an animal model. Locomotor activity was initially associated with theta oscillations (6–12 Hz) in hippocampal LFP (Kramis et al., 1975), and this rhythm was subsequently shown to be prominent in hypothalamic locomotor regions that were functionally identified as locomotion-inducing sites (Sławińska and Kasicki, 1995, 1998). Similar LFP activity might be expected of other locomotor areas of the brain, because theta oscillatory rhythms in LFPs are thought to coherently bind cooperating neuronal ensembles to produce behaviors, including locomotion (Bland and Oddie, 2001). However, LFPs have never been recorded during spontaneous locomotion from MLR sites functionally defined by stimulation to evoke locomotion in the same animal, although these sites are clearly a component of the ensemble of brain areas controlling locomotion.

Gamma band activity has been proposed to be a characteristic feature of the PPN (Garcia-Rill et al., 2015), and the intrinsic properties of PPN neurons recorded *in vitro* (Simon et al., 2010; Garcia-Rill et al., 2011, 2015) have led to the suggestion that their discharge in the gamma range of frequencies “…explains the requirement to stimulate this region at 40–60 Hz to optimally induce locomotion” (Garcia-Rill et al., 2015). However, clinical studies using DBS have indicated that lower frequencies of stimulation are more effective (Ferraye et al., 2010; Nosko et al., 2015). Optogenetic activation of the MLR at frequencies lower than 40 Hz has recently been shown to reliably induce locomotion in mice (Lee et al., 2014). Moreover, Ferraye et al. (2010) concluded that “The best effects were seen in the patients with active contacts located slightly posterior to the pedunculopontine nucleus, in the cuneiform and subcuneiform nuclei…” Microelectrode recordings have suggested that neurons that modulate firing in response to imagined gait actually tend to be located in the subcuneiform region dorsal to the PPN (Piallat et al., 2009). Thevathasan et al. (2012) showed in patients with Parkinsonism that alpha power (frequency range equivalent to our theta in rats) was maximal in the caudal PPN region, and they demonstrated a correlation between alpha oscillations and improved gait performance. This caudal “pedunculopontine region” corresponds to the cuneiform and subcuneiform nuclei in humans (Ferraye et al., 2010; Alam et al., 2011). Thus there is increasing evidence that the emphasis on the PPN as the most effective site for DBS to improve locomotion may not be warranted (Alam et al., 2011), and there is increasing evidence for the CnF involvement in human studies instead. We undertook a study focusing on the CnF area of the MLR in the rat model to determine the LFP signature of this area in recordings from confirmed DBS sites for eliciting locomotion.

Takakusaki et al. (2005, 2016) and Takakusaki (2008, 2013) have demonstrated that the CnF is effective for eliciting locomotion in decerebrate cats, while the PPN controls muscle tone and stimulation in this region may actually suppress locomotion. Other workers have challenged the importance of the PPN as a component of the MLR since PPN lesions do not alter locomotor capability (Winn, 2006; Hernández-Chan et al., 2011; MacLaren et al., 2014; Gut and Winn, 2015, 2016). Moreover, neurons activated during locomotion (indicated by the presence of the activity-dependent marker Fos) are found in the CnF and other nearby areas, including the deep mesencephalic nucleus (DpMe; Jordan, 1998; Vianna et al., 2003; Heise and Mitrofanis, 2006), but not in the PPN. PPN cholinergic neurons of the MLR have been implicated in the initiation of locomotion (reviewed in Ryczko and Dubuc, 2013). However, activation of the cholinergic component of the PPN using a chemogenetic approach induced only subtle effects on locomotion in freely moving, control rats (Pienaar et al., 2015). Takakusaki et al. (2016) have shown that the effective MLR sites are not co-extensive with the cholinergic PPN neurons in cats. A recent optogenetic study in the mouse has also cast doubt on the importance of cholinergic neurons within the PPN for the initiation of locomotion from a standstill (Roseberry et al., 2016). Rather, they have demonstrated that cholinergic neurons may play a role in the modulation of ongoing locomotion. Furthermore, they found that within the MLR the glutamatergic subpopulation encodes locomotor state and speed and is necessary and sufficient for locomotion. Taken together, these studies increase the importance of determining the LFP signature of effective MLR sites, especially those in the CnF region.

In this study, we specifically targeted the CnF region to provide the first analysis of LFPs recorded from this portion of the functionally defined MLR in intact, freely moving rats and to determine the LFP signature and the most effective stimulus parameters of these effective MLR sites. We then used recordings from these sites to test the hypothesis that the theta rhythm that is a characteristic of locomotion in other structures such as the hippocampus and the hypothalamic locomotor areas (Sławińska and Kasicki, 1995, 1998) is also prominent in the MLR during voluntary locomotion in these animals, consistent with the suggestion that the theta rhythm might bind cooperating neuronal ensembles to produce locomotion. Here we focus primarily on the theta LFP oscillations, but we also subject other frequency bands to similar analysis. We also show that the same LFP features that are prominent in intact animals persist after incomplete SCI, and suggest that they might be used as a biomarker of effective sites for electrode placements for DBS.

## Materials and Methods

Experiments were performed on 28 adult female Sprague-Dawley rats (240–350 g) following their acclimation to housing, handling and treadmill locomotor assessment. Rats were housed separately. Animals were exposed to a 12-h light/dark cycle and had free access to food and water. The number of animals used, and their pain and distress were minimized. Experimental procedures were approved by the University of Miami IACUC committee in accordance with NIH guidelines (National Institutes of Health Publications, No. 80-23; revised 1996). Experiments in Poland were carried out with the approval of the First Ethics Committee for Animal Experimentation according to the principles of experimental conditions and laboratory animal care of the European Union and the Polish Law on Protection of Animals Used for Experiments.

### MLR-DBS and EMG Electrode Implantation

Parylene-insulated, tungsten microelectrodes, (A-M Systems Inc., Carlsborg, WA, USA; 127 μm diameter, 12 degree tip taper, adjusted to 0.07–0.4 MΩ) were stereotaxically implanted into the region of the MLR under isoflurane anesthesia (Milner and Mogenson, 1988). After exposing the cranium with a short skin incision, a small ~1 mm diameter hole was drilled through the left side of the skull above the stereotaxic target (~0.7–1.2 mm anterior to the interaural line, 2.0 mm lateral to the midline). Small, stainless steel, self-tapping screws served to anchor the electrode base to the skull. A titanium screw was inserted into the parietal bone immediately opposite the electrode insertion point to serve as an attachment for the return lead (anode). The microelectrode assembly was lowered using a micromanipulator aiming for a site ~6.2 mm DV (Paxinos and Watson, 1998). The base of the microelectrode assembly was then rigidly fixed to the skull and to the screws using dental cement (Stoelting, Wood Dale, IL, USA). The wound was closed by sutures around the implant. Pin connectors projecting vertically from the electrode assembly allowed electrical connection to the voltage and/or current stimulators and to preamplifiers for recording of LFPs (see below).

For the electromyography (EMG) recordings, bipolar electrodes were implanted bilaterally in the soleus (Sol) muscle (extensor, active during the stance phase of the step cycle) and the tibialis anterior (TA) muscle (flexor, active during the swing phase of the step cycle). The electrodes were made of Teflon-coated stainless steel wire (0.24 mm in diameter; AS633, CoonerWire Co., Chatsworth, CA, USA). The tips of the electrodes with 1–1.5 mm of the insulation removed were pulled through a cutaneous incision on the back of the animal, and each of the hook electrode was inserted into the appropriate muscle and secured by a suture (Sławińska et al., 2000). The distance between the electrode tips was 1–2 mm. The connector with the other ends of the wires fixed to it, covered with dental cement (Spofa Dental, Prague, Czech Republic) and silicone (3140 RTV, Dow Corning, Midland, MI, USA), was secured to the back of the animal.

### Locomotor Threshold Determination

The current or voltage threshold for locomotor induction (minimum strength to elicit locomotor activity) was determined for all animals by stimulating awake rats in an open field. The MLR was stimulated (cathodal stimulation) during behavioral testing after recovery from surgery (~1 week) and several weeks after that in some animals to determine stability and effect of long-term implantation. Thresholds were also tested in animals subject to SCIs. Thresholds for locomotor induction were carefully tested over the stimulation range: frequency 10–70 Hz (10, 20, 50 and 70 Hz), and pulse duration 0.2, 0.5, 1.0 and 2.0 ms (Milner and Mogenson, 1988; Coles et al., 1989). During any session, a stimulation frequency was chosen and threshold strengths determined for each pulse width. Thresholds were determined in current mode (1–350 μA), but voltage assessments (typically less than 7 V) were made in some animals. Thresholds were determined by gradually increasing the strength of stimulation until locomotor movements ensued. Locomotor responses to sudden stimulation at strengths above threshold were also examined in most animals. Locomotor responses were visually classified by three experienced independent observers (FJS, CO and BRN). *Slow speed* (walk) or *Fast speed* (trot or gallop; Gillis and Biewener, 2001) was determined based on two (or more) identical observer ratings. Then, in random order, another stimulation frequency was chosen and the process was repeated. Between each pulse width test, animals were allowed to rest for approximately 30–60 s.

### LFP Recording

For the chronic LFP recordings in freely moving rats the same electrodes as for MLR-DBS stimulation were used. MLR field potential recordings were made at 2000× gain, 1–300 Hz band pass filtering, with typically a 60 Hz notch filter and sampled at 1–2 kHz for later analysis. Connection to the pre-amplifier was made via wires 60–90 cm in length attached to the pin connectors on the electrode assembly with the indifferent wire connected to the titanium screw placed in the parietal bone. Animals were placed on an adjustable speed treadmill belt enclosed by a transparent plexiglass box, with automated ventilation (Treadmill Simplex II, Columbus Instruments, Columbus, OH, USA). Animals were previously trained to voluntarily walk on the treadmill and did not receive any electrical shocks for motivation. Video of each session was recorded using a 60 Hz frame rate (3-D PeakMotus Motion Measurement System). Field potentials were recorded for 30 s periods at rest and at each treadmill speed (10, 15, 20 m/min). The order varied depending on how cooperative the animals were during the different recording sessions. In most cases, animals started from rest and the belt speed was increased incrementally to each designated speed and then the belt was turned off. In other cases, recordings commenced once stable locomotion at higher speeds was attained and then the belt speed was decreased to other speeds and then stopped. Field potentials were also recorded following SCI in some animals (up to 17 weeks). For voluntary locomotion on a treadmill in these animals, speeds were dependent upon the severity of the injury.

### Spinal Cord Injuries

Mid-thoracic contusion injuries were made using the New York University Impactor device (Gruner, 1992) under isoflurane anesthesia (2% in O_2_) and sterile technique. A dorsal laminectomy was performed on the T8 vertebra to expose the dura over the T9 spinal segment. The adjacent vertebrae were clamped to stabilize and support the column during impact. Mild, moderate and severe contusion injuries were made by dropping a 10-g rod onto the exposed spinal cord from a height of 6.25, 12.5 or 25 mm, respectively. The impact height and velocity errors were below 6%, within acceptable parameters to ensure consistency. Average compression distances of 1.283 ± 0.182, 1.653 ± 0.201 and 2.01 ± 0.187 mm for mild, moderate and severe injuries, respectively, were also in close agreement with previously published data (Basso et al., 1996). The muscles were sutured in layers and the skin was closed with stainless-steel surgical clips. Rats were allowed to recover in a temperature controlled environment and were provided with accessible water and food. Gentamicin (2–3 mg/kg; APP Pharmaceuticals, Lake Zurich, IL, USA) and buprenorphine (0.1 mg/kg; Buprenex Inj.; Reckitt Benckiser Pharmaceuticals Inc., Richmond, VA, USA) were administered after surgery for 7 and 3 days, respectively. Lactated Ringers solution was administered BID SC for 7 days (5 ml/injection). The bladder was expressed twice daily until each animal regained continence. Animals were monitored daily for skin and weight changes. Animals were removed from the study if any post-operative complications could not be successfully treated (e.g., excessive weight loss, infections and pain behaviors including self-mutilation such as autonomy or excessive grooming leading to ulceration).

### Immunohistochemistry

Under anesthesia, an electrolytic lesion was made at each stimulation site by passing a small (1 mA), brief (2–5 s) DC current through the DBS electrode. Animals were perfused intracardially with paraformaldehyde and their brains removed for histological reconstruction. The tissue surrounding the stimulation site was processed for paraffin and cut into 15 μm coronal sections. Sections were de-paraffinized and the immunohistochemical procedures optimized using a primary antibody dilution series. For the pre-adsorption control, tissue sections were incubated only with normal donkey serum (NDS: 10%; in 0.1 M phosphate buffered saline or PBS) then in 0.3% Triton X-100 in 0.1 M PBS after blocking endogenous peroxidase with 3% H_2_O_2_ at room temperature. Immunoreactivity was totally absent after omission of the primary antibodies. To examine glial scar formation around the stimulation electrode and to aid in the identification of the electrode tip within the tissue, sections were stained with an antibody to glial fibrillary acidic protein (GFAP). Sections were incubated in 1:500 rabbit polyclonal anti-GFAP (Millipore AB5804) overnight (0.1 M PBS with 0.3% Triton X-100 + 5% NDS), washed in 0.1 M PBS 0.3% Triton X-100 and then incubated in secondary donkey anti-rabbit Alexa 594 (1:200; A21207, Molecular Probes) in 0.1 M PBS with 0.3% Triton X-100 + 5% NDS. Selected sections (each in a series of 10) were then counterstained and coverslipped with either Vectashield Hard-set dry mounting media (H-1500) with 4′,6′-diamidino-2-phenylindole dilactate (DAPI) a fluorescent DNA counterstain or with Cresyl Violet to confirm anatomical localization of the electrode position and anatomical regions of the brain according to Paxinos and Watson (1998). Digital epi-fluorescent images were captured on a Zeiss 200M Axiovert microscope at 5× magnification.

### Data Analysis and Statistics

For statistical analysis of LFP activity, 15 of the tested animals were selected on the basis of a consistent and complete sequence of behavior and the absence of noise/movement artifact. The data files (digitized at 2 kHz, band-pass filtered between 1 Hz and 300 Hz) were converted to Spike2 (CED, UK) format. The spectrograms and power spectra were analyzed using built-in functions of Spike2. Movement artifacts in the LFP channels were manually removed before analysis, under visual inspection. For spectrograms a window of 2048 points was used; the calibration of the *y* axis was adjusted to the dynamics of the data and is shown on the plots. Power spectra were calculated using an FFT algorithm (Hanning window, bins/channel 2048, width of bin 0.488 Hz). The total power for the delta (0–4 Hz), low (4–6 Hz) and high (6–12 Hz) theta, beta (12–28 Hz), low (28–55 Hz) and high (65–100 Hz) gamma frequency bands and the power and frequency for the peak in the power spectra of delta and theta bands related to various locomotor speeds or resting conditions for each trial were imported to Prism 6 (GraphPad Software, Inc., La Jolla, CA, USA). The D’Agostino test was performed before statistical tests to verify that the data followed a Gaussian distribution (normality test). We chose to separate the theta band into low and high components because the theta band recorded in the hippocampus (Kramis et al., 1975) was separated into Type 1 (corresponding to our high theta and linked to locomotion) and Type II (corresponding to our low theta and associated with sleeping and immobility). Comparison of thresholds effective for inducing locomotion was performed with unpaired *t-test*. Other analyses were performed with two-way Repeated Measures ANOVA (RM ANOVA). The Holm-Sidak’s test was used for multiple comparisons. The minimum significance was set at *p* = 0.05. Linear regression analysis was performed using Prism 6 (GraphPad Software, Inc.). The speed was taken as an independent *x*-variable, while the parameters describing power and frequencies were taken as *y-variable* (see “Results” Section). The regression coefficient and the significance of slope were calculated for each analysis.

## Results

### Evoked Locomotion in Freely-Moving Animals

**Figure 1:**
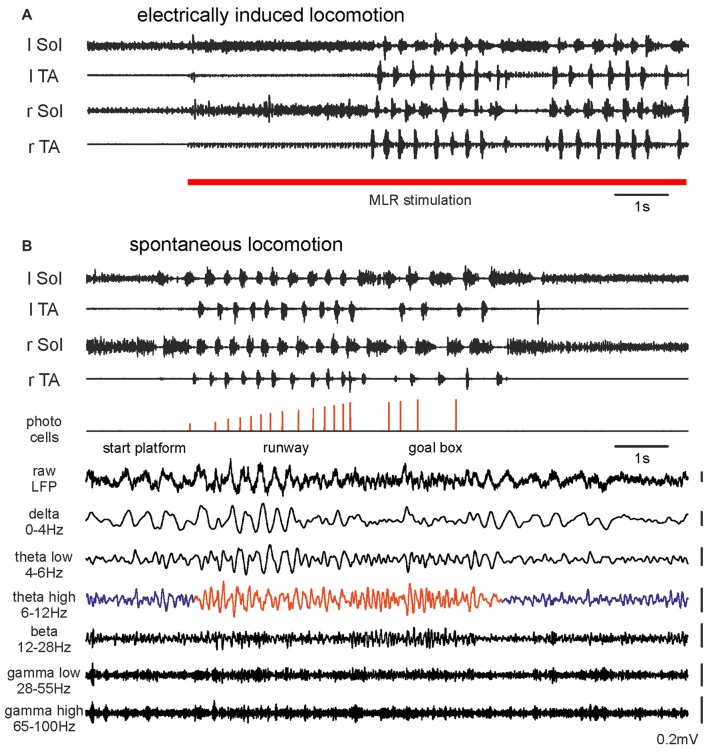
**Hindlimb muscle electromyography (EMG) and mesencephalic locomotor region (MLR) LFP activity during transition from resting to walking: (A)** A rat with motor activity induced by right MLR electrical stimulation (28 μA, 20 Hz, 0.5 ms pulse duration). **(B)** The same rat walking spontaneously along a 2 m runway. Photocells events with increasing pulse amplitudes show progression along the runway. The bottom traces show simultaneously occurring raw LFP recorded from the right MLR and successive activity in filtered frequency bands. Abbreviations: Sol, Soleus muscle; TA, Tibialis Anterior muscle; r, right; l, left; LFP, local field potential.

The efficacy of MLR stimulation was examined in freely moving animals within 1 week of implantation. Figure 1A shows an example of EMG recordings during electrically induced locomotor behavior of a rat in an open field. A stimulus applied in the MLR at a strength just above threshold (28 μA) induced increased attention indicated by an orienting reaction, looking around and then forward quadrupedal locomotion commencing around 3.5 s after the onset of the stimulation, leading to regular locomotion. This pattern of activity is similar to that observed in freely moving cats with the stimulating electrode implanted in the cuneiform nucleus (Mori et al., 1989).

Figure 1B presents EMGs and LFPs from the same effective site within the MLR during voluntary locomotion of the same rat along a runway. The animal’s movement was detected with photocells positioned at intervals along the runway. MLR LFPs recorded prior to, during and after voluntary locomotor activity on a runway showed dramatic changes during the transition from rest to walking. During recording from the effective MLR site we observed irregular LFP activity during resting. Transition to the locomotor activity detected with EMG was associated with clearly enhanced theta rhythm (6–12 Hz; Figures 1B, 4B). This change in LFPs was observed in all animals where the recording site was within an effective MLR stimulus site (Figures 3A–C). In Figure 1B we also present examples of activity in other filtered bands (delta, low and high theta, beta, low and high gamma). Changes related to locomotor activity were also observed in delta and low theta bands, but they did not persist throughout the locomotor trial, and the delta band activity could also be observed at rest. See below for more detailed analysis.

**Figure 2:**
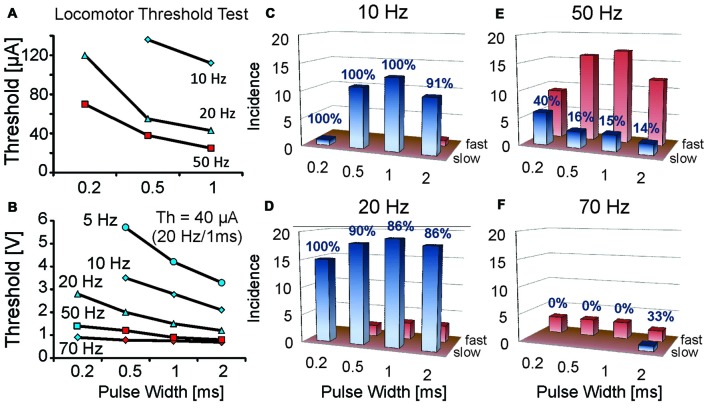
**Rate of locomotion at threshold strengths for initiating locomotion varies with different stimulation frequencies and pulse widths.** Locomotor threshold parameter tests for single MLR stimulation sites were conducted at various stimulus frequencies and pulse widths. **(A,B)** Thresholds plotted relative to pulse width and frequency for two animals; one intact **(A)** and one with mid-thoracic moderate contusion injury **(B)**. Current threshold (Th) for the spinal cord injury (SCI) animal at 20 Hz, 1 ms duration was 40 μA. Note that greater strengths of stimulation were required with lower frequencies and shorter pulse widths. Initial locomotor responses vary according to frequency and pulse width of stimulation: stimulation frequencies of 20 Hz typically produce slow locomotion at threshold (blue symbols) in contrast to higher stimulation frequencies which typically initiate fast locomotion at threshold (red symbols). **(C–F)** Incidence of slow or fast locomotion at threshold strengths for initiating locomotion for different stimulation frequencies and pulse widths in uninjured animals 1 week after electrode implant. Note higher incidence of slow locomotion (percentage indicated) at low stimulation frequencies (10 and 20 Hz) vs. high frequencies (50 and 70 Hz). The number of trials is indicated on the ordinate (1 trial per animal). Red bars indicate the incidence of fast locomotion, while the blue bars indicate the incidence of slow locomotion. Only one data point per animal is included for each frequency and pulse width test combination. A similar response was observed during threshold tests in the SCI animal **(B)**.

**Figure 3:**
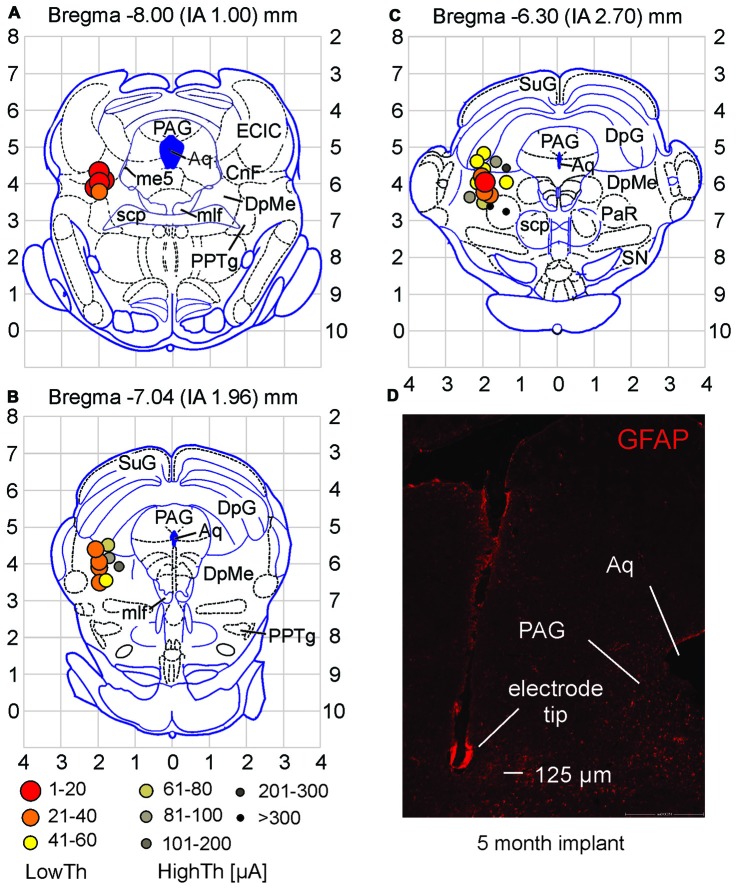
**Stimulation sites for high and low current locomotor threshold groups differ in location. (A–C)** Low threshold sites were found in the CnF and DpMe of midbrain. High threshold sites were observed surrounding these areas. Current thresholds for each site are defined by symbols in **(B)**. **(D)** Example of electrode tip site in the brain tissue (coronal section). Note the minor tissue response to the electrode as observed with GFAP staining. Abbreviations as per Paxinos and Watson (1998). Aq, aqueduct; CnF, cuneiform n.; DpG, deep gray layer of superior colliculus; DpMe, deep mesencephalic n.; ECIC, external cortex of inferior colliculus; me5, mesencephalic n. of 5th nerve; mlf, medial longitudinal fasciculus; PAG, periaqueductal gray; PaR, pararubral n.; PPTg, pedunculopontine tegmental n.; scp, superior cerebellar peduncle; SN, substantia nigra; SuG, superficial gray layer of superior colliculus.

**Figure 4:**
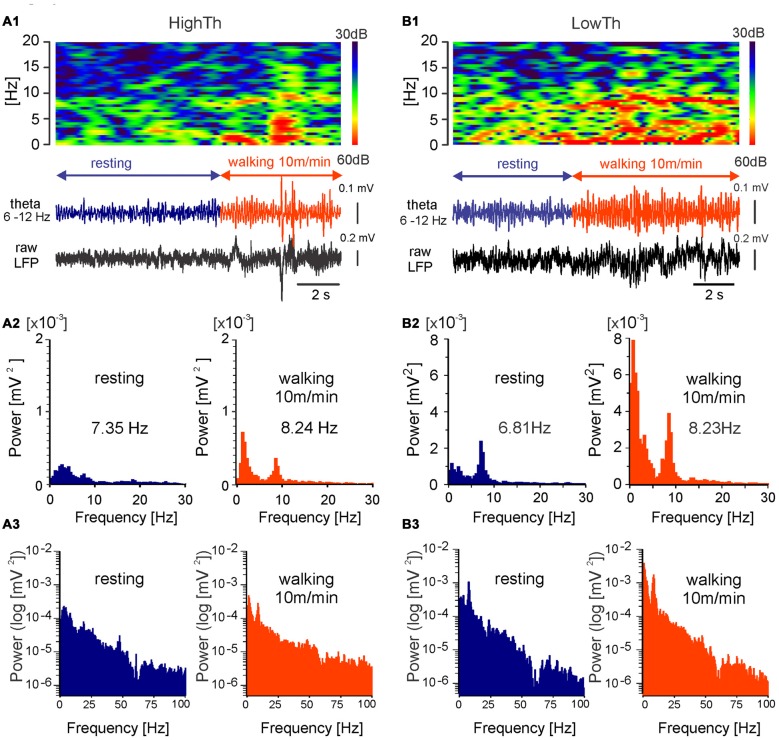
**MLR LFP activity during transition from resting to walking in freely moving rat.** LFPs recorded during locomotion from LowTh sites show a larger increase in the power of delta and theta bands in comparison to the HighTh sites. Top in **(A1)** frequency spectrogram (frequencies between 0 Hz and 20 Hz) plotted vs. time of LFP recorded from a HighTh site; bottom in **(A1)** LFP, filtered 6–12 Hz and raw, recorded from a HighTh site; **(A2)** histograms of power spectra in linear scale for corresponding data segments; **(A3)** histograms of power spectra in logarithmic scale for corresponding data segments; Top in **(B1)** frequency spectrogram (frequencies between 0 Hz and 20 Hz) plotted vs. time; bottom in **(B1)** LFP, filtered 6–12 Hz and raw, recorded from LowTh site; **(B2)** histograms of power spectra for corresponding data segments. **(B3)** histograms of power spectra in logarithmic scale for corresponding data segments.

### Stimulation Parameters for Evoked Locomotion in an Open Field

Stimulation of the MLR evoked locomotor responses ranging from slow walking to fast locomotor behavior and jumping depending upon the stimulus parameters. Stimulation strength was slowly increased during testing for locomotor threshold values. At each of the tested stimulation parameters (frequency or pulse width), when strength of stimulation approached threshold value, animals would typically orient themselves and lean forward slightly before initiating locomotion. This response could last many seconds, becoming shorter with an increase in the rate at which the stimulation strength was increased. The delay to the onset of the locomotor response was much shorter (and could be within a second) when stimulation strengths at the onset of stimulation were above threshold and the locomotor response could be abrupt (galloping with or without jumping), often resembling escape behavior.

Locomotor threshold tests over a range of frequencies (10–70 Hz) and pulse widths (0.2–2 ms) were conducted in 21 uninjured animals at ~1 week of implantation (Figure 2). Walking could be most readily produced at 10 or 20 Hz. Stimulation at and above threshold strength at these frequencies produced well-graded locomotor responses evolving from slow walking to galloping. Transition from slow to fast locomotion typically occurred over a range of stimulus strengths (10–20 μA), thus demonstrating good controllability. At 50 or 70 Hz stimulation frequencies the first locomotor response produced at threshold strength was predominantly fast locomotion. Increasing the strength of stimulation above threshold could induce galloping and jumping responses and typically with only slight changes in strength of stimulation (2–5 μA). Tests in SCI animals (*n* = 3) showed a similar frequency dependence of the locomotor response as uninjured animals at threshold stimulation strengths (Figure 2B). Thus, a “controlled” rate of locomotion was difficult to establish at the highest tested frequencies, in contrast to 20 Hz, which produced the best controllable, graded locomotor response.

### High vs. Low Locomotor Threshold Sites

Since the most consistent controllable locomotion was obtained at a frequency of 20 Hz with pulse width of 0.5–1 ms (Figure 2B), we determined the threshold strengths at approximately 1 week after implantation using 1 ms pulses at 20 Hz and grouped the animals into those with either high or low threshold responses. We defined low threshold (LowTh) sites as those producing locomotion with currents of ≤60 μA and those above 60 μA were defined as high threshold (HighTh).

Locations of all electrode stimulation sites (*n* = 28) were plotted on three coronal planes centered on Bregma −8.00, −7.04 and −6.30 mm (interaural 1.00–2.70 mm; taken from the atlas of Paxinos and Watson, 1998). High and low current locomotor threshold groups differed in location within the midbrain (Figures 3A–C). Low threshold sites were found in the CnF and the DpMe more anteriorly. High threshold sites were observed surrounding these areas dorsally, medially and ventrally.

GFAP staining was observed along each electrode tract and especially at the tip. This is illustrated in Figure 3D taken from an animal with a 5 month implant. GFAP staining typically did not extend into surrounding tissue for more than 50 or 60 μm even after prolonged implantation. To determine whether prolonged implantation of the electrode and increased glial scar formation (He and Bellamkonda, 2008) would affect locomotor thresholds (at 20 Hz, 1 ms pulse duration), we re-tested thresholds in 19 animals at various times (9–116 days) following the initial threshold assessment. Using current controlled stimulation, eight animals showed increased current thresholds, while nine showed decreased thresholds and two were unchanged. Overall, current thresholds were not significantly different (*p* = 0.342; paired samples *t-test*) with time from implantation, indicating that the small deposition of GFAP around the electrode tip did not interfere with electrode stimulation efficacy.

### LFP Recorded from MLR-DBS Sites during Treadmill Locomotion

The chronic LFP recordings in freely moving rats were obtained using electrodes that were implanted for DBS. Analysis of LFPs was done for periods before and during forced locomotion at various speeds (10, 15 and 20 m/min) of the treadmill belt. Examples of the LFPs (filtered within the theta range, 6–12 Hz) recorded from a HighTh and a LowTh site in rats during transition from resting to walking are shown in Figures 4A1,B1 (bottom). In contrast to rats of the HighTh group, in all rats of the LowTh group, a regular theta rhythm was present from the start to the end of locomotion. Before locomotion started, short lasting episodes of regular activity in the LFP with a peak frequency of about 6–7 Hz were recorded (spectrogram in Figure 4B2—left). Interrupting these were short episodes of rhythmic LFP activity with a peak frequency below 7 Hz that were usually related to exploratory behavior of the rat in the treadmill cage. When the rat started to walk a clear peak of rhythmic theta activity (7–9 Hz) appeared in the spectrogram. A comparison of the power spectra calculated for the LFP recorded during resting and locomotion shows that the different experimental conditions differ in both frequency and amplitude of the peak in the power spectra of the theta band (see example in Figures 4A2,B2). There was also a change in the peak in the power spectra of the delta band (0–4 Hz; see analysis below and Figure 7). The data presented on the logarithmic scale show no peak in LFP power in the higher frequency bands (Figures 4A3,B3).

**Figure 5:**
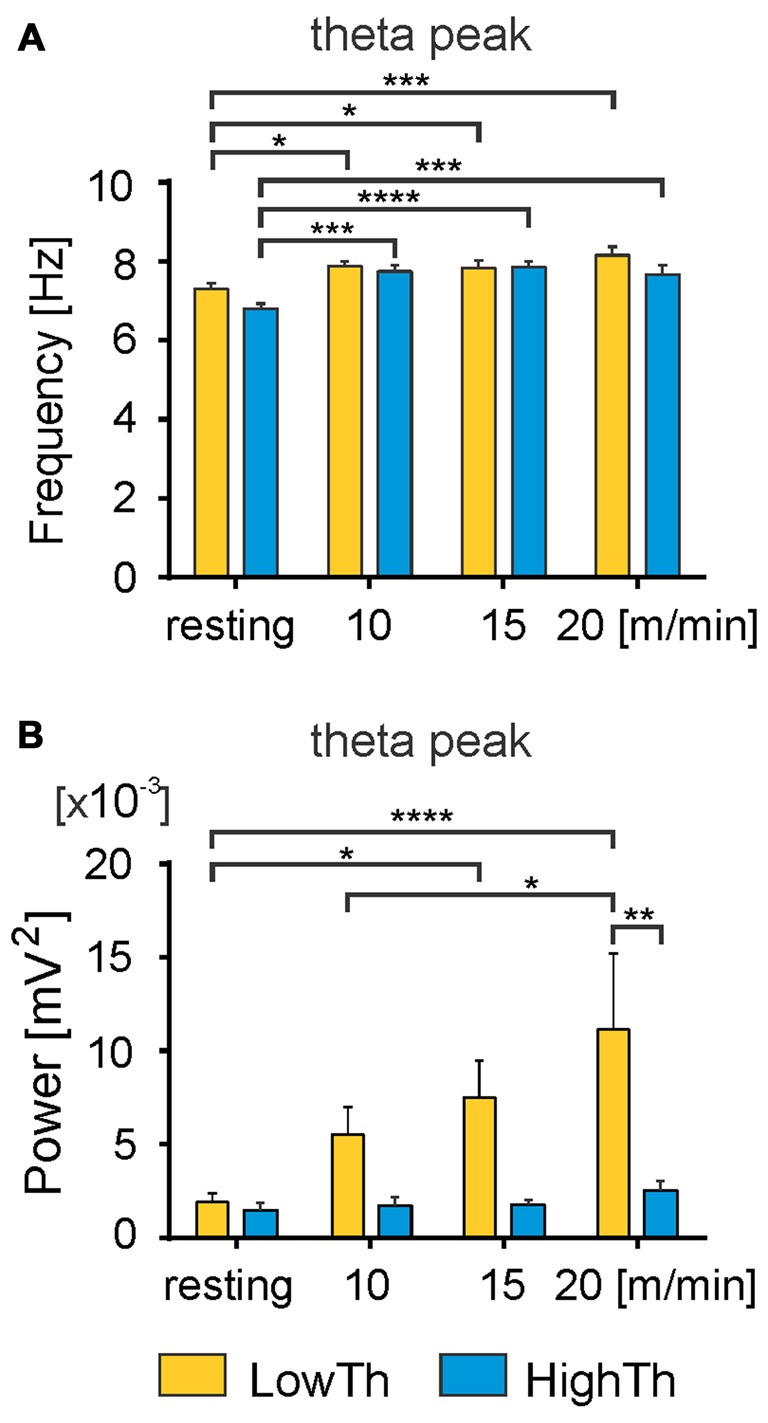
**Changes in frequency and power of the theta peak with the transition from resting state to locomotion.** A significant increase in the frequency of theta peak was observed with the transition from resting to locomotion in both LowTh and HighTh animals **(A)**. Note significant increase in power of the theta peak in the LowTh group between resting and locomotion at 15 m/min and 20 m/min, and between 10 m/min and 20 m/min **(B)**. The power of the theta peak was significantly different when comparing LowTh and HighTh rats during locomotion at the highest speed (20 m/min). *P < 0.05, **P < 0.01, ***P < 0.001, ****P < 0.0001.

**Figure 6:**
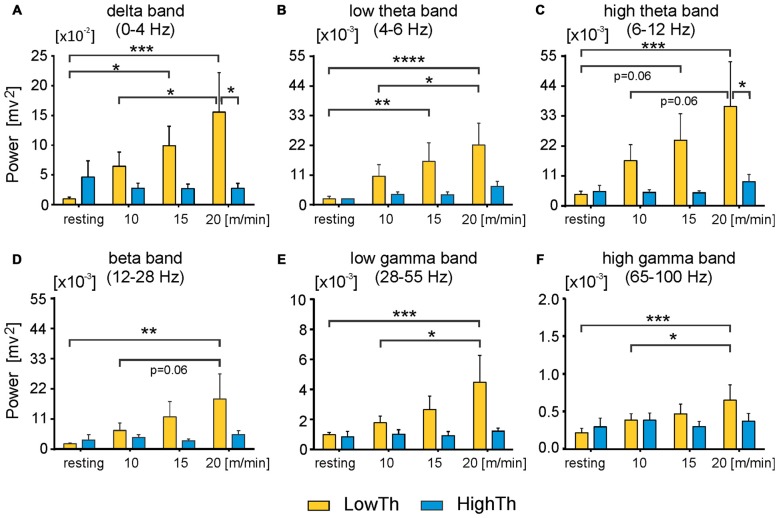
**Power of all analyzed frequency bands in LFPs significantly increases in low threshold MLR sites but not high threshold sites as locomotor speed increases.** Power of delta **(A)** low and high theta band (**B,C** respectively) and beta band **(D)** significantly increases in LowTh sites, but not HighTh sites as voluntary locomotor speed increases. **(E,F)** Power of low and high gamma band in LowTh sites also shows significant increases as locomotor speed increases. Note, difference in scaling between individual plots for power of different bands. Power of any band is the integral of the power in the specific frequency ranges: delta: 0–4 Hz; theta low: 4–6 Hz; theta high: 6–12 Hz; beta: 12–28 Hz; gamma low: 28–55 Hz; gamma high: 65–100 Hz (*n* = 8 LowTh; *n* = 7 HighTh). *P < 0.05, **P < 0.01, ***P < 0.001, ****P < 0.0001).

**Figure 7:**
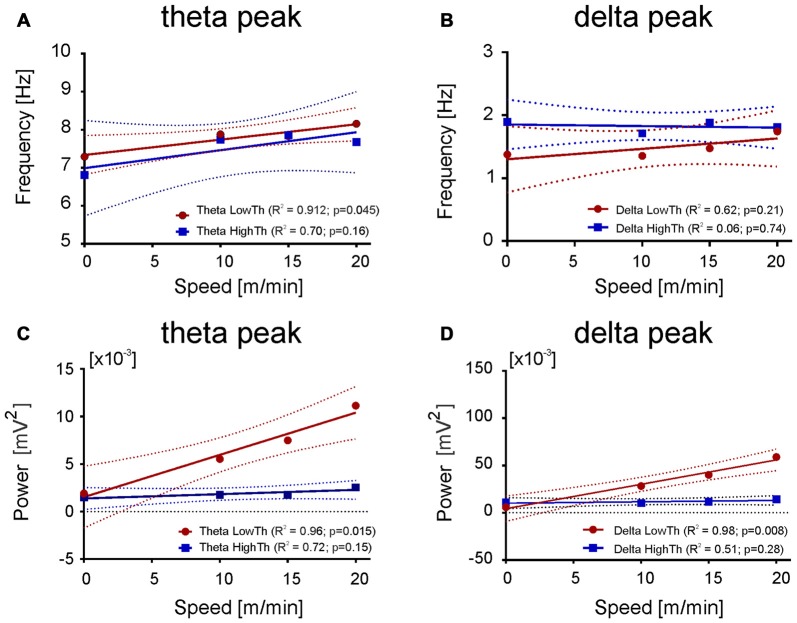
**Relationship between the frequency and power of maximum in the delta and theta band and the speed. (A,C)** Both frequency and power of theta peak increase with the speed of an animal for LFPs recorded from the LowTh sites, while for the HighTh sites the relationship was not significant. **(B,D)** Opposite to that the frequency of delta peak was not related with the speed of rat both for LowTh and HighTh sites. Note that in contrast to the theta peak, in the delta peak only the power was related to the speed of locomotion of the animals of LowTh group only.

We performed an analysis comparing the frequency of the peak of theta rhythm and its power between the LowTh and HighTh groups. We selected eight LowTh and seven HighTh cases for statistical analysis based on the fact that they were all subjected to the protocol for LFP recordings at rest and at all defined treadmill speeds (10, 15 and 20 m/min). Average threshold currents for these LowTh and HighTh sites were 32.8 ± 3.2 and 151.5 ± 38.3 μA and were significantly different (*t*-test, *p* ≤ 0.001). Statistical analysis (two-way RM ANOVA) showed a significant increase in frequency of the peak of theta rhythm over the resting condition at all speeds of locomotion in both groups of animals (*F*_(3,39)_ = 16.34, *p* < 0.0001; Figure 5A). Interestingly, within each experimental condition (rest or locomotion) the differences of the frequency of the theta oscillations between the LowTh and HighTh groups were not significant.

Analysis of the peak power of the theta rhythm (Figure 5B) showed significant differences between resting and locomotion at 15 m/min and 20 m/min, and between 10 m/min and 20 m/min in the LowTh group only (*F*_(3,39)_ = 5.075, *p* < 0.005). In the HighTh group we found no significant differences between any of the experimental conditions. Thus, it is clear that the theta rhythm was present both during rest and locomotion and the frequency of the theta rhythm was not different, but the peak power of the theta rhythm differentiated the LowTh and HighTh groups. It is noteworthy that the peak power of the theta oscillations was significantly different (Sidak’s test, *p* = 0.0066) when comparing LowTh and HighTh rats during locomotion at the highest speed (20 m/min; Figure 5B). In summary, our results show that both the frequency and peak power of the theta rhythm change depending on the locomotor speed of animals in the LowTh group, while in the HighTh group only the frequency of the peak of the theta rhythm changes. This shows that theta oscillations are associated with sites that are most effective for eliciting locomotion. In line with this result is a conclusion presented by Thevathasan et al. (2012) who found that in PD patients the best outcome of DBS was achieved at the level of maximal alpha activity in the caudal PPN region.

Next, we analyzed the differences in power of delta, theta, beta and gamma bands between the LowTh and HighTh groups for the different experimental conditions (Figure 6). For the theta band we analyzed two sub-bands: low (4–6 Hz) and high (6–12 Hz) theta; for gamma we analyzed low (28–55 Hz) and high (65–100 Hz) sub-bands.

For the LowTh group a two-way RM ANOVA did not show significant differences between experimental conditions for the delta band (*F*_(3,39)_ = 2.739, *p* = 0.056). *Post hoc* Sidak’s multiple comparison tests showed that power was significantly different between resting and locomotion at 15 m/min and between 10 m/min and 20 m/min locomotion (Figure 6A). There were no significant differences between the different behavioral states in the HighTh group. Moreover, the difference between LowTh and HighTh groups for locomotion at 20 m/min was significant (Figure 6A).

For the LowTh group a two-way RM ANOVA showed significant differences between experimental conditions for the low theta band (*F*_(3,39)_ = 6.642, *p* = 0.001) and for the high theta band (*F*_(3,39)_ = 3.790, *p* = 0.0177). *Post hoc* Sidak’s multiple comparison tests showed that power was significantly different between resting and locomotion for both bands of theta at speeds of 15 m/min and 20 m/min, and between 10 m/min and 20 m/min (Figures 6B,C). There were no significant differences between the different behavioral states in the HighTh group. Moreover, the difference between LowTh and HighTh groups for locomotion at 20 m/min was significant (Figure 6C).

For the power of the beta band, the differences between various behavioral states (i.e., resting and locomotion at different speeds) were significant (*F*_(3,39)_ = 3.071, *p* = 0.0389) only in the LowTh group (Figure 6D). The power observed at rest was significantly different from that observed during locomotion at 20 m/min (Sidak’s test, *p* = 0.0026).

For the low gamma band we found significant differences between various behavioral states (*F*_(3,39)_ = 4.189, *p* = 0.0116). *Post hoc* analysis showed differences between resting and locomotion at 20 m/min, and locomotion at speeds of 10 m/min and 20 m/min (Figure 6E) only in the LowTh group. The same results were obtained for the high gamma band (*F*_(3,39)_ = 4.203, *p* = 0.0114; Figure 6F). These results show that there are progressive changes for all frequency bands as locomotion increases in speed.

Results shown in Figure 6 suggested the possibility of a relationship between the power of frequency bands and the speed. Thus, we performed linear regression analysis which showed that changes in power of delta, theta, beta and gamma bands are related to the speed changes (data not shown). As the relation between speed and the power of delta and theta bands were much stronger (for delta *R*^2^ = 0.96, *p* = 0.016; for theta high *R*^2^ = 0.97, *p* = 0.015) than for the other bands and as in both bands there is characteristic peak of frequency visible, we decided to analyze their parameters further, i.e., the frequency and power of the peak frequency in relation to the speed of locomotion. Figure 7 shows the regression lines for LowTh and HighTh groups. For the peak of the power spectra of the theta band the changes of both the frequency (Figure 7A) and power (Figure 7C) were positively related to the speed in the LowTh group (slopes of both regression lines were significantly different from zero (*p* < 0.05 and *p* < 0.02, respectively). For the peak of the delta band such a relationship was significant only for the power (slope significantly different from zero at *p* < 0.05) but not for the frequency of the delta peak (Figures 7B,D). This suggests that the theta rhythm represents a more meaningful biomarker for effective MLR sites.

**Figure 8:**
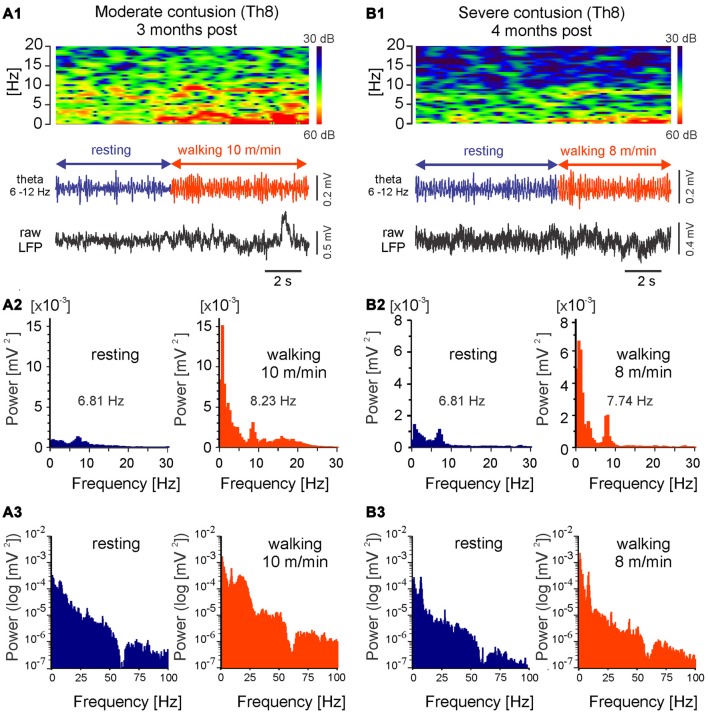
**LFPs recorded from LowTh sites in rats with moderate or severe mid-thoracic contusion SCI show similar correlation to locomotor speed as intact animals.** Top in **(A1,B1)**: frequency spectrogram plot vs. time; bottom in **(A1,B1)**: LFP, filtered 6–12 Hz and raw; **(A2,B2)**: histograms of power spectra in linear scale; **(A3,B3)**: histograms of power spectra in logarithmic scale. Note increased power within the theta band (<12 Hz) and shift in the dominating theta frequency with the transition to walking.

Because the LFP frequency is a potential means of localizing the most effective sites for DBS for therapeutic purposes, we also determined whether the theta rhythm in LFPs persists after SCI. As Figure 8 illustrates, in rats after chronic SCI the theta oscillations can be observed in MLR LFP spectrograms and power spectra during locomotion, similar to those we have described for intact rats. These characteristics of LFP that we describe here can therefore be considered reliable indices of the location of potentially effective MLR stimulus sites in injured animals. These LFP characteristics were observed in all of the rats with SCI with low threshold MLR sites (*n* = 8) included in this article. In each of these animals, DBS improved locomotor activity. A full analysis of the effect of MLR stimulation on locomotor activity observed following contusive SCI will be presented elsewhere.

## Discussion

### LFPs in Functionally Defined MLR in Intact Rats Display a Characteristic Theta Rhythm during Voluntary Locomotor Activity

This is the first observation of LFP activity recorded in freely moving rats from functionally identified locomotion-inducing MLR sites. We found that the onset of voluntary locomotor activity was invariably accompanied by a transition from disorganized activity to a prominent theta rhythm. This is in accordance with previous studies on hypothalamic locomotor sites that were functionally identified (Sławińska and Kasicki, 1995, 1998). This is also the typical LFP frequency band observed in hippocampal CA1 at bipolar recording sites during locomotion (Kramis et al., 1975; Bland, 1986). Our data confirm our hypothesis that the theta rhythm is a characteristic of LFP activity in the MLR during voluntary locomotion, and are consistent with the suggestion that the theta rhythm serves to link different cell groups together in functional ensembles (Tort et al., 2008; Colgin, 2016).

The transition from resting to locomotion was associated with increases in the power spectra. For two bands, delta and theta, the increase was clear. Moreover, in both bands clear peaks were visible. Thus, we did a more thorough analysis for those bands. Namely, we analyzed the frequency and power of the peak frequency in both bands. For the delta band, we found that the frequency of the peak was not related to the speed for either the LowTh or the HighTh group. The power of the delta peak frequency was related to the speed for the LowTh group only. Thus, we can conclude that the changes in delta band parameters are not locomotion related. We also analyzed the relationship of the theta rhythm to the speed of locomotion. There are several differences between theta band parameters when comparing the LowTh vs. HighTh groups. The power of the theta band was significantly greater (by up to 5-fold) in the LowTh animals than in the HighTh groups. Furthermore, the power and the frequency of the peak theta frequency was related to the speed of locomotion in the LowTh group of animals, but not in the HighTh group, analogous to our previous finding, where we showed theta recorded from the hippocampus was related to the speed of locomotion (Sławińska and Kasicki, 1998). We consider these facts as further support for the suggestion that locomotion is associated with theta rhythm in structures implicated in the control of locomotion, including the MLR.

Our finding that the peak in the power spectrum is highly correlated with the speed of locomotion in the LowTh cases suggests that the theta oscillations may be a reliable biomarker for electrode placement in an effective MLR site for inducing locomotion. We are aware of the potential for signal contamination due to the use of monopolar recording, but we think this is an unlikely confound. The fact that the power of the theta peak is significantly larger in the LowTh cases than in the HighTh cases that are spatially more remote from the most effective site clearly demonstrates that the LFPs recorded in these cases are generated locally and not due to volume conductance. The placement of our reference electrode also argues against volume conduction from the hippocampus, because it was substantially caudal to the region of the hippocampus, and the hippocampus was not between the reference and the LFP electrodes.

The relationship of our theta LFPs (the peak frequency and power) to speed of locomotion is noteworthy, because this is typical of other areas in the brain implicated in locomotor control, including the hypothalamus and the hippocampus. Theta oscillations in the hippocampus depend on the activity of the medial septal neurons, and the speed of locomotion is conveyed to the hippocampus via glutamatergic septohippocampal projections (King et al., 1998; Fuhrmann et al., 2015). Optogenetic stimulation at theta frequencies of axons of medial septal GABAergic cells in the hippocampus via an optic fiber implanted above the CA1 area induced theta oscillations but did not initiate locomotion in immobile mice (Bender et al., 2015), while optogenetic activation of the medial septal glutamatergic input to the hippocampus induced locomotion and hippocampal theta rhythm (Fuhrmann et al., 2015). However, both articles described that theta oscillations were correlated with the speed of locomotion. The means whereby the hippocampal activity can modulate or initiate locomotion is not known, but either the hypothalamic or mesencephalic locomotor regions may be involved. This is an issue that needs clarification. It is of particular interest because hippocampal theta oscillations are thought to be involved in encoding the animal’s position during spatial navigation (Bender et al., 2015). The presence of a locomotor speed-related theta rhythm in the MLR is consistent with the hypothesis that these areas are coherently bound together during locomotion. Perhaps the purpose of such binding might be to preserve position and speed information throughout the locomotor system.

The presence of similar theta rhythms in multiple sites related to locomotion (MLR, hypothalamic locomotor regions and hippocampus) is consistent with functional coupling of these structures. Although Bland and Vanderwolf (1972) showed electrical stimulation of the hippocampus does not induce locomotion, recent experiments showed chemogenetic or optogenetic activation of hippocampal cells (including CA1 neurons) resulted in enhanced locomotor behavior (Alexander et al., 2009; Fuhrmann et al., 2015). In the chemogenetic study, Alexander et al. (2009) expressed HA-hM3Dq driven by the CaMKIIa promoter in mice and found that the otherwise inert ligand for hM3Dq, clozapine-N-oxide (CNO), markedly increased locomotor activity. These findings are consistent with the well-known coupling between the limbic and motor systems (Mogenson and Nielsen, 1984), and with functional coupling of locomotor regions through a common LFP frequency as previously proposed (Sławińska and Kasicki, 1995).

The persistence of this characteristic theta oscillation at sites that promote locomotor recovery after SCI suggests that it can be used as a biomarker in clinically relevant circumstances, and can guide that placement of DBS electrodes for effective restoration of locomotion in injured subjects. Further studies are needed to determine the impact, if any, of damage or degeneration in cases such as partial SCI on the MLR locomotor system. There is evidence of plasticity in multiple descending pathways after SCI (Ballermann and Fouad, 2006; Filli et al., 2014; Leszczyńska et al., 2015; Fink and Cafferty, 2016; Hansen et al., 2016), and changes in brainstem locomotor circuits are possible. However, our results so far provide a clear indication that the factors producing theta oscillations in the MLR during even impaired locomotion after partial SCI persist to the degree that the potential biomarker role for theta oscillations is maintained.

A predominance of gamma band activity in the MLR might be expected based on the activity of single PPN neurons *in vitro* (Garcia-Rill et al., 2015). However, in contrast to those results, no other peak in higher frequency bands (including gamma) was visible in the power spectra in the rats included in our study. This is consistent with the observation that the firing of single PPN neurons during actual locomotion was less than 6 Hz in normal rats (Geng et al., 2016).These same authors reported LFP results from PPN during locomotion consistent with our findings from effective MLR sites, with the highest percentage of relative LFP power observed in the 0.7–12 Hz range. Their PPN sites were not confirmed to be effective locomotion-inducing sites, however. And their PPN recording sites were anatomically distinct from our MLR sites (more ventral). The much lower power of beta and gamma bands also varied for different experimental conditions (treadmill speed) in the LowTh group but no such variations occurred in the HighTh animals. The frequency of the theta rhythm increased significantly as the animal started to walk. This is consistent with the association of the theta rhythm at these sites with the processes underlying locomotion. These findings have the potential to aid in the identification of brainstem sites that should be the most effective for DBS facilitation of locomotion, providing for the first time a theta rhythm signature for the MLR. Emerging clinical data is consistent with this. According to Tattersall et al. (2014), LFP recordings from the supposed PPN area of the MLR have shown oscillations in both the alpha (6–12 Hz) and beta (12–30 Hz) frequency bands (Weinberger et al., 2008; Shimamoto et al., 2010; Tsang et al., 2010; Thevathasan et al., 2012; Tattersall et al., 2014). Active gait was also accompanied by increased LFP alpha power in this area (Thevathasan et al., 2012; Tattersall et al., 2014). Recordings from DBS electrodes implanted in the PPN area (PD patients) showed an increase of the power in the range 7–11 Hz (the alpha range in human, which overlaps with the high theta; 6–12 Hz in our article) after symptom improvement with levodopa administration (Androulidakis et al., 2008). It was also shown in PD patients after levodopa that LFPs recorded from the PPN area showed “an increase in PPNa alpha (5–12 Hz) oscillatory activity and a decrease in beta (13–35 Hz) and gamma (65–90 Hz) bands activity” (Fraix et al., 2013).

Like most areas of the brain, the MLR region receives input in a range of frequencies. The arrangement of MLR neurons, the orientation of their dendritic fields, and the organization of the synaptic input could create more synchronous LFPs or they could cancel out others in the MLR area. Thus neurons in MLR may be receiving significant gamma frequency input, for example, but because of the microanatomy of local circuits, synchronized extracellular current flow may be minimal in the gamma range. However, we were unable to find evidence for an endogenous rhythm in the gamma range, as suggested for the PPN (Simon et al., 2010; Garcia-Rill et al., 2011).

### Effective Stimulus Sites and Parameters for Inducing Locomotion in Freely-Moving Rats

Low threshold MLR sites (below 60 μA, mean 32.8 μA) were found in the CnF and the more rostral DpMe. This is consistent with recent findings from human studies using DBS for treatment of FOG, where it has been suggested that the most effective targets are within the CnF and subcuneiform regions instead of the PPN (Piallat et al., 2009; Alam et al., 2011). This conclusion is in agreement with many previous animal studies (Shik et al., 1966; Ross and Sinnamon, 1984; Steeves and Jordan, 1984; Milner and Mogenson, 1988; Coles et al., 1989; Mori et al., 1989; Jordan, 1998; Takakusaki et al., 2016). A recent trans-synaptic tracing study also implicates the CnF (Xiang et al., 2013). Although there are a number of studies implicating the PPN as the effective site for DBS in humans (reviewed in Collomb-Clerc and Welter, 2015; Hamacher et al., 2015; Nonnekes et al., 2015; Udupa and Chen, 2015), it is widely recognized that the effective sites are difficult to clearly identify (Alam et al., 2011). fMRI of the brainstem in human subjects shows the CnF is activated during imaginary gait (Karachi et al., 2012). Nevertheless, it is common in the clinical literature to equate the PPN with the MLR, and the suggestion has been made that the term MLR can now be “retired” (Garcia-Rill et al., 2015). But Hernández-Chan et al. (2011) and Winn and co-workers (Winn, 2006; Gut and Winn, 2015, 2016) have pointed out weaknesses in the argument that the PPN is an essential component of the MLR, and these arguments along with our data support the notion that a functional definition of the MLR is still valid. Takakusaki et al. (2005, 2016) and Takakusaki (2008, 2013) have directly compared the effects of CnF and PPN stimulation, and they found that the CnF is effective for eliciting locomotion in decerebrate cats, while the PPN controls muscle tone, and stimulation in this region may actually suppress locomotion. Also, they showed the effective MLR sites are not co-extensive with cholinergic neurons that define the location of PPN. Moreover, neurons activated during locomotion (indicated by the presence of the activity-dependent marker Fos) are found in the CnF and other nearby areas, including the DpMe (Jordan, 1998; Vianna et al., 2003; Heise and Mitrofanis, 2006), but not in the PPN. Stimulation in the DpMe area also induces locomotion in the rat (Melnikova, 1975; Milner and Mogenson, 1988; Coles et al., 1989; Cabaj et al., 2017) and does not harbor cholinergic neurons.

PPN cholinergic neurons have been considered essential for MLR initiation of locomotion (Garcia-Rill and Skinner, 1987) in mammals and lamprey (reviewed in Ryczko and Dubuc, 2013). However, recent data does not support a critical role for a cholinergic component of the MLR in the initiation of locomotion. Activation of the cholinergic component of the PPN using a chemogenetic approach induced only subtle effects on locomotion in freely moving, control rats (Pienaar et al., 2015), and optogenetic stimulation of cholinergic neurons in the PPN did not elicit locomotion in stationary mice (Roseberry et al., 2016). Glutamatergic rather than cholinergic neurons of the MLR have been shown to be effective for eliciting locomotion with optogenetic stimulation (Lee et al., 2014; Roseberry et al., 2016). Furthermore, cholinergic antagonists do not impair the initiation of locomotion due to electrical stimulation of the MLR (Jordan et al., 2014). Taken together these data increase the likelihood that components of the MLR other than the PPN should be the target for DBS to restore locomotion.

In clinical DBS studies, the electrode size and methods of detecting electrode placement do not provide sufficient resolution to rule out the possibility that the stimulus activates structures outside of the anatomically defined PPN. As a result the term PPNa, for the area of the brainstem surrounding the PPN, including CnF and precuneiformis, has been introduced (Fraix et al., 2013; Welter et al., 2015). Further research in animal models is required using modern methods of activating and silencing genetically defined putative MLR constituents to clarify this issue, but the continued use of the term PPN instead of MLR to describe effective sites for locomotion induction is misleading.

We determined the most effective stimulation parameters for inducing controllable locomotion (pulses of 1.0 ms duration at 20 Hz). These are relevant to the selection of stimulation parameters for the treatment of gait defects using DBS, but are in contrast to the previous claim, based upon results from PPN stimulation in decerebrate rats and recordings from PPN neurons *in vitro*, that stimuli in the 40–60 Hz range should be required (Garcia-Rill et al., 2015). Our results show that the effective frequency for electrical stimulation is not predicted by the peak frequency of LFP recorded during locomotion in the same area, but must be determined empirically. We contend that the definition of “optimal stimulation frequency” should necessarily take into consideration the ability to induce controllable locomotion, i.e., to “dial-in” locomotor speed and maintain it. According to this definition and in these targeted sites, stimulation frequencies lower than gamma are optimal. In keeping with this, DBS experience for gait improvement in PD has led to the conclusion that the effective frequency of stimulation is often in the range of 10–25 Hz instead of higher frequencies (Androulidakis et al., 2008; Ferraye et al., 2010, 2011; Nosko et al., 2015). Others have also shown that stimulation in the MLR at the lower frequency ranges (5–30 Hz) was suitable for inducing locomotion in adult rats (Melnikova, 1975; MacDonell et al., 2015) and cats (Douglas et al., 1993; Guertin et al., 1995; Noga et al., 2003).

### Concluding Remarks

Our results are consistent with earlier observations that the onset of locomotion is characterized by theta frequency oscillations of the LFPs in hippocampus and in locomotion-inducing sites in the subthalamic locomotor region and posterior hypothalamus (Sławińska and Kasicki, 1995, 1998). We suggest that theta oscillatory LFPs may coherently bind cooperating neuronal ensembles during locomotor activity in order to encode the animal’s position during spatial navigation. It is clear from our results that after injury the theta rhythm can also be used as a signature for effective sites for DBS, especially as DBS in the MLR dramatically improved locomotion in animals with a SCI.

## Author Contributions

LMJ, US and BRN: conceptualization; BRN, LMJ, US and SK: methodology; BRN, FJS, CO, LMV, US, MO, AMC and HM: investigation; BRN, US and SK: formal analysis; BRN and US: visualization; BRN, LMJ, US and SK: writing—original draft; BRN, LMJ, US and SK: writing—review and editing; BRN and US: supervision; BRN, US, SK and LMJ: funding acquisition.

## Conflict of Interest Statement

The authors declare that the research was conducted in the absence of any commercial or financial relationships that could be construed as a potential conflict of interest.
